# Cell-free DNA screening for aneuploidies in 7113 pregnancies: single Italian centre study

**DOI:** 10.1017/S001667232000004X

**Published:** 2020-06-16

**Authors:** Alvaro Mesoraca, Katia Margiotti, Claudio Dello Russo, Anthony Cesta, Antonella Cima, Salvatore Antonio Longo, Maria Antonietta Barone, Antonella Viola, Davide Sparacino, Claudio Giorlandino

**Affiliations:** 1 Human Genetics Lab, Altamedica Main Centre, Viale Liegi 45, 00198 Rome, Italy; 2 Department of Prenatal Diagnosis, Altamedica, Fetal–Maternal Medical Centre, Viale Liegi 45, 00198 Rome, Italy

**Keywords:** cell-free foetal DNA, cffDNA, chromosome aneuploidies, next-generation sequencing, NIPT

## Abstract

**Introduction:**

Non-invasive prenatal testing (NIPT) using cell-free foetal DNA has been widely accepted in recent years for detecting common foetal chromosome aneuploidies, such as trisomies 13, 18 and 21, and sex chromosome aneuploidies. In this study, the practical clinical performance of our foetal DNA testing was evaluated for analysing all chromosome aberrations among 7113 pregnancies in Italy.

**Methods:**

This study was a retrospective analysis of collected NIPT data from the Ion S5 next-generation sequencing platform obtained from Altamedica Medical Centre in Rome, Italy.

**Results:**

In this study, NIPT showed 100% sensitivity and 99.9% specificity for trisomies 13, 18 and 21. Out of the 7113 samples analysed, 74 cases (1%) were positive by NIPT testing; foetal karyotyping and follow-up results validated 2 trisomy 13 cases, 5 trisomy 18 cases, 58 trisomy 21 cases and 10 sex chromosome aneuploidy cases. There were no false-negative results.

**Conclusion:**

In our hands, NIPT had high sensitivity and specificity for common chromosomal aneuploidies such as trisomies 13, 18 and 21.

## 1. Introduction

Until recently, prenatal screening for foetal aneuploidies relied on measurement of maternal serum biochemical markers such as serum *α*-fetoprotein (AFP) or *β*-human chorionic gonadotropin (*β*-hCG) combined with an ultrasound test. The discovery of cell-free foetal DNA (cffDNA) in maternal plasma prompted the development of non-invasive prenatal testing (NIPT), introducing into clinical practice a new approach for the screening of common foetal aneuploidies, reducing unnecessary invasive procedures such us amniocentesis and chorionic villus sampling that may result in miscarriage or intrauterine infection (Odibo *et al.*, 2008). NIPT is based mainly on targeted and whole-genome-based technologies employing next-generation sequencing (NGS). These technologies rely on the ability to detect increases in cffDNA arising from the presence of an extra foetal chromosome. The clinical introduction of NIPT NGS has been successfully reported in many clinical validation studies (Sehnert *et al.*, 2011; Norton *et al.*, 2012; Nicolaides *et al.*, 2013; Verweij *et al.*, 2013; Bianchi *et al.*, 2014; Pergament *et al.*, 2014), showing a detection rate of more than 99%, with a foetal fraction of less than 1% for trisomy 21 (Gil *et al.*, 2014). According to a systematic review and meta-analysis, the pooled sensitivity was 97.4% for trisomy 13, 97.4% for trisomy 18 and 99.3% for trisomy 21 (Taylor-Phillips *et al.*, 2016). NIPT clinical services were introduced in Italy in 2012, but a large-scale clinical study is still lacking. The objective of this study was to evaluate the overall clinical performance of our foetal DNA NIPT-NGS-based methodology in detecting trisomies 13, 18 and 21 and sex chromosome aneuploidies (SCAs) in a cohort of 7113 samples.

## 2. Materials and methods

### 2.1. Subjects

The study includes the retrospective investigation of 7113 pregnant women who were admitted at Altamedica Medical Centre (Rome, Italy) and underwent foetal cffDNA screening tests between January 2018 and March 2019. The present study was approved by the local Ethics Committee of Artemisia SPA and all participating women provided their written informed consent. In this cohort, confirmation feedback was available for positive sample results.

### 2.2. Sample collection and NIPT analysis

In total, 7113 cases were tested using the Ion S5 NGS (ThermoFisher Scientific, Waltham, MA, USA) platform. The features of the cases and their indications for NIPT are summarized in Table 1.

**Table 1.** Patient characteristics of 7113 pregnancies undergoing non-invasive prenatal testing (NIPT) for chromosomal aneuploidy.

Clinical characteristics	Mean value (range)
Maternal age	33.4 (20–50)
Body mass index (kg/m^2^)	23.2 (14.5–43.5)
Gestational age (weeks)	16.5 (10–26)
**Indications for NIPT**	**Ratios (%)**
Maternal serum screening result	26.3%
Advanced maternal age ≥35 years	41.2%
Others (family history, *in vitro* fertilization pregnancy, decision of perinatology council, etc.)	32.5%

The following protocol was used: blood samples were collected in Streck Cell-Free DNA BCT tubes (Streck, La Vista, NE, USA) and sample were centrifuged on the same day. Approximately 3–4 mL of plasma was isolated and stored at –80°C until cffDNA extraction. The cffDNA was extracted using the Qiasymphony DSP Virus or Qiasymphony Circulating DNA Kit (Qiagen, Valencia, CA, USA). The DNA concentration was determined using the Agilent Technologies 4200 TapStation System (Agilent Technologies, Santa Clara, CA, USA) and the High Sensitivity D1000 ScreenTape System (Agilent Technologies). The acceptable cffDNA range for processing the sample was between 80 and 120 pg/μL. DNA libraries were prepared using the Ion Ampliseq Kit according to the manufacturer's instructions (ThermoFisher Scientific). The resulting libraries were sequenced using the Ion S5 system. The results of the tests were provided to patients by post-test counselling; invasive diagnosis was offered for positive reports. Generally, invasive testing methods such as quantitative fluorescent polymerase chain reaction and karyotyping by amniocentesis were selected in order to confirm the results.

### 2.3. Data analysis

Sequencing data were analysed using a proprietary algorithm developed by Life&Soft Company (Plessis-Robinson, France). Briefly, the detection of foetal aneuploidies is based on a combination of high-quality alignments against the human genome and read counts to identify chromosomal gains. A first normalization is performed based on GC percentage to calculate a high-quality z-score. A second normalization is performed in each sample to compute another z-score based on a chromosome not involved in the aneuploidy detection. The presence of a foetal aneuploidy for all 24 chromosomes is assessed using the values of the two z-score calculations. A lack of a result in a sample was attributed to an insufficient (<4%) fraction of cffDNA or failure to pass the quality control measures. Based on these results, chromosomal aneuploidy was determined in the foetus. The sensitivity, specificity and positive predictive value (PPV) were calculated using the VassarStats online calculator (http://www.vassarstats.net).

## 3. Results and discussion

The objective of this study was to evaluate the performance of our foetal DNA test in detecting trisomies 13, 18, and 21 and SCAs in a cohort of 7113 high-risk or intermediate-risk pregnancies arriving in our laboratory between January 2018 and March 2019. The median gestational age in this testing cohort was 16.5 weeks (Table 1). The median maternal age was 33.4 years. The median body mass index was 23.2 kg/m^2^. The median foetal fraction of reported samples was 7.3%. In this cohort, twin pregnancy samples represented 0.8% of all pregnancies tested. The median time for reporting was 5 business days. Repeat samples were reported in 1.6% (119/7113) of the sample cohort, and these were due to insufficient foetal fraction (below a pre-specified threshold of 4%), low reads and discordant sex. In this cohort, the overall frequency for autosomal aneuploidies was 0.9% for trisomies 13, 18 and 21 collectively, while the frequency of reported SCAs was 0.14% (Table 2). In order to assess the accuracy of the positive NIPT results, follow-up based on confirmatory testing using amniocentesis for abnormal samples is recommended. Follow-up confirmation results were available for 3 samples with trisomy 13, 7 samples with trisomy 18, 62 samples with trisomy 21 and 13 samples with SCAs, reported as ‘Confirmed’ in Table 2. Data on pregnancy outcome were missing for five cases, in particular two for trisomy 18, one for monosomy X, one for trisomy X and one for 47, XYY karyotypes, all reported as ‘Unconfirmed’ in Table 2. Missing data were due to loss of contact or because the women declined follow-up. Based on the confirmatory follow-up, the PPVs indicating the probability that a foetus with a positive NIPT test truly has the genetic disorder were 66.7% for trisomy 13, 71.4% for trisomy 18 and 93.5% for trisomy 21 (Table 2). Since all negative NIPT cases were found to be negative based on this outcome, we assume the mutation detection rate of our test to be close to 100%. Follow-up results for SCAs were obtained for nine samples with monosomy X (Turner syndrome), two samples with trisomy X, two samples with 47, XXY (Klinefelter syndrome) and no samples with 47, XYY. Estimated PPVs were 66.7% for monosomy X and 100% for trisomy X and 47, XXY (Table 2). Among 7113 pregnant women who underwent NIPT analysis, we diagnosed 90 cases with abnormal NIPT results. Of these, 75 cases were confirmed to be true-positive results with PPVs of 66.7%, 71.4% and 93.5% for trisomies 13, 18 and 21, respectively, and PPVs of 66.7% for monosomy X and 100% for trisomy X and 47, XXY, while 5 cases could not be considered further due to a lack of communication with the patients (Table 2).

**Table 2.** Foetal DNA clinical performance.

Chromosome aneuploidies	NIPT test				
Total	7113				
		**Confirmed**			
Normal (negative)	7023	7023			
Trisomies 21, 13 and 18 (positive)	74				
False-negative trisomy 21	0				
False-negative trisomy 13	0				
False-negative trisomy 18	0				
		**Confirmed**	**Specificity (%)**	**Sensitivity (%)**	**PPV (%)**
Trisomy 21	62	62	99.9 (95% CI = 99.8–100%)	100 (95% CI = 92.3–100%)	93.5 (95% CI = 83.5–97.9%)
True positive	58				
False positive	4				
Unconfirmed	0				
Trisomy 13	3	3	99.9 (95% CI = 99.9–100%)	100 (95% CI = 19.8–100%)	66.7 (95% CI = 12.5–98.2%)
True positive	2				
False positive	1				
Unconfirmed	0				
Trisomy 18	9	7	99.9 (95% CI = 99.9–100%)	100 (95% CI = 46.3–100%)	71.4 (95% CI = 30.3–94.9%)
True positive	5				
False positive	2				
Unconfirmed	2				
NIPT results for SCA	16				
Monosomy X	10	9	100 (95% CI = 99.9–100%)	100 (95% CI = 51.7–100%)	66.7 (95% CI = 30.9–91.0%)
True positive	6				
False positive	3				
Unconfirmed	1				
Trisomy X	3	2	100 (95% CI = 99.9–100%)	100 (95% CI = 19.8–100%)	100 (95% CI = 19.8–100%)
True positive	2				
False positive	0				
Unconfirmed	1				
47, XXY	2	2	100 (95% CI = 99.9–100%)	100 (95% CI = 19.8–100%)	100 (95% CI = 19.8–100%)
True positive	2				
False positive	0				
Unconfirmed	0				
47, XYY	1	0	–	–	–
True positive	0				
False positive	0				
Unconfirmed	1				

CI = confidence interval; NIPT = non-invasive prenatal testing; PPV = positive predictive value; SCA = sex chromosome aneuploidy.

cffDNA screening for aneuploidies by NGS-based methodologies has been widely used for trisomies 13, 18 and 21 in recent years, but clinical studies on its efficacy at single centres are lacking. Foetal DNA testing is a NIPT test with high performance that is able to detect common autosomal trisomies and SCAs such as monosomy X, 47, XXY, trisomy X and 47, XYY. Our test results were SCA positive in 16 samples, and 10 samples were validated by karyotyping, showing an overall true-positive rate of 63%. This study has a number of limitations for SCA analysis. First, due to the low incidence of SCAs in the general population (1/400 new-borns) more pregnancies must be evaluated in order to better investigate and define the accuracy of this test for the detection of SCA syndromes. Secondly, since new-borns with SCA syndromes can appear phenotypically normal, the negative predictive value could not be calculated, and so caution needs to be expressed in these types of studies, unless all neonates undergo karyotyping analysis.

Nevertheless, in our population, the PPV was higher than or similar to that reported by others (Yao *et al.*, 2014; Bianchi *et al.*, 2015). Petersen *et al*. (2017) found PPVs of 26% for monosomy X, 50% for trisomy X and 86% for 47, XXY.

Our prospective study collected 7113 clinical samples and evaluated the NIPT strength by analysing the cytogenetics or phenotypic outcomes. In conclusion, our study represents a clinical experience of NIPT in Italy where prenatal screening by this method is used predominantly as a secondary screening test. We have shown that the performance of NIPT in detecting trisomies 13, 18 and 21 was maintained at a high level, comparable to that of a recently validated clinical study (Kypri *et al.*, 2019). This study offers further proof of the high degree of accuracy of NGS-based NIPT analysis compared to conventional screening methods.
